# Seed viability and germination success of *Acacia tortilis* along land‐use and aridity gradients in the Eastern Sahara

**DOI:** 10.1002/ece3.1851

**Published:** 2015-12-29

**Authors:** Gidske Leknæs Andersen, Knut Krzywinski, Håkon K. Gjessing, Richard Holton Pierce

**Affiliations:** ^1^ UNI Research EnvironmentP O Box 78105020BergenNorway; ^2^ Department of GeographyUniversity of BergenP O Box 78025020BergenNorway; ^3^ Department of BiologyUniversity of BergenP O Box 78005020BergenNorway; ^4^ Norwegian Institute of Public HealthP O Box 4404 Nydalen0403OsloNorway; ^5^ Department of Global Public Health and Primary CareUniversity of BergenBergenNorway; ^6^ Department of Linguistic, Literary and Aesthetic StudiesUniversity of BergenSydnesplassen 75007BergenNorway

**Keywords:** Body‐mass, bruchid infestation, camel, dung, nomadic pastoralism, ovicaprids, ruminant herbivores, seed dormancy

## Abstract

Our study focuses on the keystone species *Acacia tortilis* and is the first to investigate the effect of domestic ungulates and aridity on seed viability and germination over an extensive part of the Eastern Sahara. Bruchids infest its seeds and reduce their viability and germination, but ingestion by ruminant herbivores diminishes infestation levels and enhances/promotes seed viability and germination. The degree of these effects seems to be correlated with animal body mass. Significantly reduced numbers of wild ruminant ungulates have increased the potential importance of domestic animals and pastoral nomadism for the functionality of arid North African and Middle Eastern ecosystems. We sampled seeds (16,543) from *A. tortilis* in eight areas in three regions with different aridity and land use. We tested the effect of geography and sampling context on seed infestation using random effects logistic regressions. We did a randomized and balanced germination experiment including 1193 seeds, treated with different manure. Germination time and rates across geography, sampling context, and infestation status were analyzed using time‐to‐event analyses, Kaplan–Meier curves and proportional hazards Cox regressions. Bruchid infestation is very high (80%), and the effects of context are significant. Neither partial infestation nor adding manure had a positive effect on germination. There is a strong indication that intact, uningested seeds from acacia populations in the extremely arid Western Desert germinate more slowly and have a higher fraction of hard seeds than in the Eastern Desert and the Red Sea Hills. For ingested seeds in the pastoralist areas we find that intact seeds from goat dung germinate significantly better than those from camel dung. This is contrary to the expected body‐mass effect. There is no effect of site or variation in tribal management.

## Introduction

**Figure 1:**
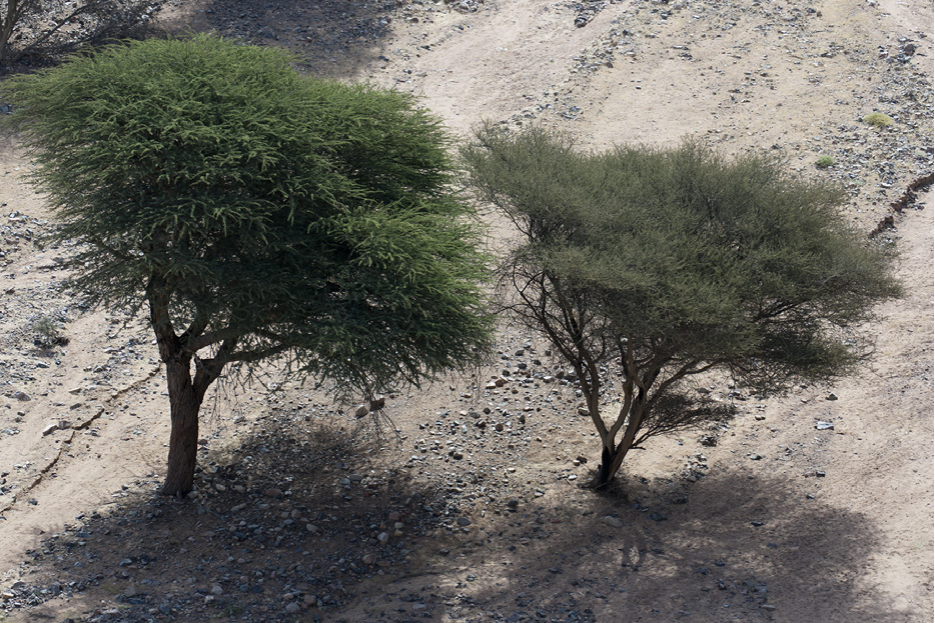
*Acacia tortilis* (Forssk.) Hayne supsp. *raddiana* (Savi) Brenan (left) is the dominant of the two subspecies of *A. tortilis* in the study area; subsp. *tortilis* is seen to the right.According to Kyalangalilwa et al, 2013 the newly formalised official name for *Acacia tortilis* is *Vachellia tortilis* (Forssk.) Galasso & Banfi.

The woody perennial species *Acacia tortilis* (Forssk.) Hayne (Fig. 1) is distributed over a vast territory across a wide range of gradients of altitude and moisture and exhibits a particular adaptability to arid and hyperarid conditions. It is a dominant biological and cultural keystone species and a vital resource for nomadic pastoralists (Andersen et al. 2014; Hobbs et al. 2014). Current evidence suggests this important species exhibits high mortality and low recruitment, which threatens both regional biodiversity and the livelihoods of people dependent upon it (Ward and Rohner 1997; Andersen and Krzywinski 2007).

*Acacia tortilis* survival depends on the recruitment of new individuals, which requires the presence of viable, nondormant seeds to respond to rare instances of optimal rainfall (Wilson and Witkowski 1998; Rohner and Ward 1999). Larval infestation by bruchid beetles seriously threatens the presence of viable seeds because larvae destroy very many seeds (Janzen 1969; Halevy 1974; Miller 1996a; Rohner and Ward 1999; Or and Ward 2003; Ward et al. 2010). However, large mammalian herbivores mitigate risk (by dispersal) and negative effects (by ingestion) of infestation, and hence positively affect seed viability and germination (summarized by Or and Ward 2003). These positive effects appear to be positively correlated with the body mass of ruminant herbivores (retention time) and their tooth size (small animals destroy more intact seeds) (Miller and Coe 1993; Miller 1995; Rohner and Ward 1999).

At best germination in variable environments is risky, and high postgermination seedling mortality is normal and a main limiting factor for long‐term species survival (Rohner and Ward 1999). However, a prerequisite for entering the recruitment game is intact seeds. This is secured both by the abundance of seeds produced by many woody perennial Leguminosae species (Janzen 1969) and the mitigating and positive effects of ruminants on bruchid infestation. For annual plants, delayed germination is a known reproduction strategy (Cohen 1966), but little is known about possible adaptations and strategies of seeds of woody perennials in extreme deserts.

Most studies focusing on seed viability, bruchid infestation, and germination of *A. tortilis* have been on small spatial scales and have not taken into account regional variation in land use and aridity (Or and Ward 2003). Our study is the first to address viability and germination over a wide area. The deserts of Egypt and eastern Sudan cover the most arid part of the distribution range of *A. tortilis* including several isolated populations (Darius 2013). Domesticated animals have been present there since the origin of nomadic pastoralism (Bubenzer et al. 2007), and both are still locally present. This is, however, rapidly changing (Hobbs et al. 2014). While it has been suggested that domestic animals, particularly camels, are important for the conservation of acacias in the Middle East (Rohner and Ward 1999), no studies have investigated the relative effect of ovicaprids and camels on bruchid infestation and germination. This article attempts to quantify the scope of the bruchid infestation in *A. tortilis* seeds sampled across the Eastern Sahara and to test germination across a land‐use and aridity gradient. We explore effects of bruchid infestation, manure, and ingestion/body mass on germination. In light of the new information gained we also assess delayed germination as a survival strategy for *A. tortilis* during extreme aridity.

## Materials and Methods

### Study area

**Figure 2:**
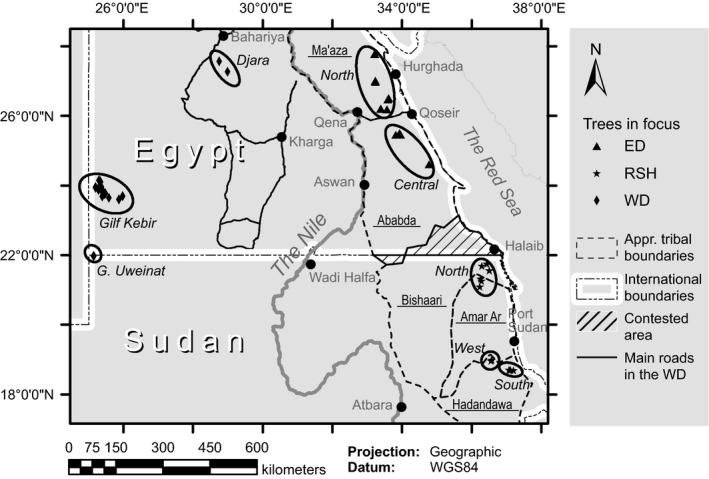
Map of study area showing the *regions* (ED: Eastern Desert, RSH:Red Sea Hills, and WD: Western Desert) and distribution of trees in focus within *areas,* outlined by ellipses (ED: North and Central; RSH: North, West, and South; WD: Djara, Gilf Kebir, and G. Uweinat), and tribal territories (underlined names) in ED and RSH (note that the contested area is mainly within Bishaari territory).

Within the Eastern Sahara we collected seeds from eight areas within three different regions representing gradients in aridity and land‐use: the hyperarid Western (WD) and Eastern (ED) Deserts of Egypt and the arid Sudanese Red Sea Hills (RSH; Fig. 2). Rainfall is extremely low and infrequent, exhibiting high spatiotemporal variability, and details are poorly known because there are hardly any meteorological stations. The dominant tree species within all regions is *Acacia tortilis* Forssk. (Hayne) subsp. *raddiana* (Savi) Brenan but *A. tortilis* subsp. *tortilis* also grows in parts of the ED and RSH (Boulos 1999) (Fig 1). Stands of trees of both subspecies are often widely separated in *wadis* (dry river valleys) intersecting mountainous landscapes. These wadis occasionally flood after torrential rainfall, and subsurface water there is sufficient for sustaining populations and/or single trees.

Rainfall in the WD (<5 mm/year) does not recur annually. Seasonally, rain is more common in winter, but may fall any time of year (Darius 2013). In its southwestern corner, the mountain massif of G. Uweinat (Fig. 2) attracts orographic rain from monsoonal winds due to its altitude (peak about 1900 m asl.), and mean annual rainfall is closer to 10 mm. Except for G. Uweinat which has warm winters (mean of coldest month 20–30°C), the WD has mild winters (mean of coldest month 10–20°C but frost can occur) and very hot summers (mean temperature of hottest month >30°C; Ayyad and Ghabbour 1985).

In the mountainous deserts east of the Nile mean annual rainfall ranges from around 10 mm in the north (mainly winter rain) to around 100 mm in the south (both winter and monsoonal summer rain). East of the mountain range in the RSH, summers are hot (mean temperature of hottest month 20–30°C) and winters mild, while the areas to the west have warm winters (Ayyad and Ghabbour 1985).

Seeds from the WD were collected from three main *areas* (Fig. 2). Two isolated populations (41 km apart) were sampled in the Djara area (rainfall c. 3 mm/year, only winter) located on the Egyptian limestone plateau. Perennial vegetation is extremely rare there. The Gilf Kebir plateau (GK) and G. Uweinat (UWT) mountain are in the southwestern corner of the WD (Fig. 2) surrounded by extensive sand bodies. Populations of *A. tortilis* subsp. *raddiana* grow in some wadis in the sandstone massif of GK (1100 m asl; rainfall c. 5 mm/year, any season, very high evapotranspiration) and in the mainly granitic UWT. Presently, all these areas lack human habitation, but previously there have been episodic visits of both nomads and camel caravans (Bubenzer et al. 2007). Today GK and UWT in particular are destinations for scientists and tourists (e.g., Bubenzer et al. 2007; however completely closed for legal visitors since autumn 2014) and stopovers for camel caravans from Sudan to Kufra, Libya, and for poachers and smugglers. Wild ruminant ungulates in the region include populations of *Ammotragus lervia* Barbary sheep, *Gazella dorcas* Dorcas gazelle, and *Capra nubiana* Nubian ibex.

Pastoral nomadic tribes are still present in *areas* east of the Nile (Fig. 2). In the ED seeds were collected from the Ma'aza tribal area in the north (EDN) and in the central Ababda tribal area (EDC). In the RSH, we collected seeds from the Amar Ar and Bishaari tribal areas in the north (RSH_N), and in the southern (RSH_S) and western part (RSH_W) within the Hadandawa tribal area. The northern areas of the ED, and in particular Ma'aza area, are more affected by sedentarization and abandonment (Hobbs et al. 2014) although domestic animals still roam there. In parts of RSH, particularly more remote areas, traditional nomadism is still practiced (see Andersen et al. 2014; Hobbs et al. 2014). Domestic animals include camel, goat, and locally some sheep. Wild ruminant ungulates include *G. dorcas* and *C. nubiana*, although also here poaching has decimated populations.

Key characteristics for sites are summarized in Table 1.

**Table 1:** Areas and regions summarized according to some key environmental and land‐use characteristics

Region	Area	Aridity	Dominant tribe	Trad. Land use	Other human impacts	Tree populations	Tree density
ED	EDN	Drier	Ma'aza	Mainly abandoned	Tourism	Few due to deforestation up to the 1950s	Very sparse
	EDC		Ababda	Transition toward abandonment		Found in most wadis	
RSH	RSH_N	Dry	Bishaari	Mainly active	Tourism rare	Found in most wadis	Sparse
	RSH_W		Hadandawa				
	RSH_S						
WD	Djara	Driest	–	Completely absent	Caravans, Limitedtourism (area closed since autumn 2014)	Trees found only in isolated populations or as single individuals	Very sparse
	GK		–			Isolated populations found in some wadis only	
	UWT		–				Very sparse ‐ sparse

### Material

Seeds were collected between April 2010 and November 2011 according to a multilevel sampling design (Table 2). At the lowest level sampling focused on *contexts* linked to randomly selected individuals of *A. tortilis* supsp. *raddiana*. We collected separate samples preferably from three different contexts: (1) Canopy (only in WD, GK, and UWT) with *subcontext*: green pod, dry pods, black pods (only used in WD); (2) Ground with *subcontext*: loose seeds, green pod, dry pod, or (3) Dung with *subcontext*: camel or ovicaprid (most probably goat; Barbary sheep and ibex dung was indistinguishable from goat/sheep dung). Gazelle dung contained no seeds, possibly because seeds were sorted out during rumination (see Miller 1995). Dung pellets can have contained seeds from both subspecies.

**Table 2:** Overview of the multilevel sampling design according to the three regions. “Trees in focus” refers to the three different contexts sampled from and around randomly selected trees

Region	ED	RSH	WD
# Areas	2	3	3
# Sites	9	10	7
# Trees in focus	22	20	42

Fresh/dry fruits were sampled from a canopy until a 1‐L bag was full. From the ground surface, under a given tree's canopy, a similar amount was sampled for each of contexts 2 and 3 within a chosen square meter. In some cases, this was not sufficient and sampling time/area was extended. Samples from all contexts are interlinked through the variable *tree ID*, which represents a randomly select tree. Seeds from context 2 and 3 may derive not only from the selected tree but also from the nearby tree population. The *tree ID* variable thus represents a selected tree and its close proximity.

Fieldwork could not always be scheduled to the season when pods ripen on the tree (timing of ripening vs. personnel and permit restrictions). We therefore sampled from what could be found in the three different contexts. This reflects a natural situation where what is available will constitute the current seed source and germinate, or not, when rain falls.

All sample‐bags were frozen (<−20°C, min 48 h) before preliminary sorting and preparation at Department of Biology, University of Bergen. The dung pellets were carefully broken up and the seeds collected. All seeds were categorized individually as to bruchid infestation by *Status*: intact/uninfested (H0), 1 entry hole (H1), 2 or more holes (H2), destroyed, and sorted according to sub‐/context.

### Seed viability

To investigate factors determining the degree of seed infestation we studied the risk of having at least one hole, using a logistic regression on all seeds sampled. The outcome (dependent) variable was thus dichotomous (0: no holes; 1: at least one hole). Predictors were the categorical variables subcontext and area. The logistic regression estimates the odds ratio (OR) of infestation. The odds of infestation are defined as the probability of infestation divided by the probability of noninfestation, and the OR is the odds in a given area divided by the odds in a reference area. At the regional level RSH was used as reference, at the area level RSH_N was used, at context level dung, and at subcontext dry pods on the ground.

It is a standard assumption of logistic regression that measurements are independent. However, as one would expect there is a substantial amount of overdispersion, that is, that an area around a tree would have either a large number of beetle infestations or very few, depending on the presence of beetles in the area. To correct for this, a random effects logistic regression allows the infestation rate to vary substantially among trees selected ‐including their immediate surroundings‐ by including *tree ID* as a random effect (Rabe‐Hesketh and Skrondal 2012). The analyses were performed using the lme4 package (Bates et al. 2014) in the statistical programming environment R (2014).

### Germination experiment

For the germination experiment, we selected seeds from the full seed assemblage. This facilitated comparison of seed germination across geographical region, context, and infestation status. To maximize statistical power within an acceptable total sample size, seeds were thus selected to ensure sufficient coverage of all subgroups to be compared, that is, as close to a balanced design as possible within the constraints of the original seed collection. This was done by selecting all seeds in the smallest subgroups and selecting at random a sufficient number of seeds from the others. In total, 1193 seeds were selected, which was deemed sufficient to statistically detect differences in germination related to tested effects: bruchid infestation (see Material); manure (see below); animal body mass (dung/subcontext); site (Table 2); and tribal management (Table 1).

Each seed was then randomized to be planted either in wet soil only, or together with manure from camel or goat, with approximately equal numbers in each category. Finally, seeds were assigned a random serial number that specified the sequence in which they were planted. This ensured that all factors to be studied in the experiment were independent of time of planting, position in plant rows, available sunlight etc.

The experiment was performed at the Red Sea University, Port Sudan, starting February 20th, 2012. According to data from GHCND (Menne et al. 2012) the mean temperature for Port Sudan in the period from 1957–2013 is 23, 24, and 27°C for February, March, and April, respectively. Optimal germination temperature for *A. tortilis* seeds is 25°C (Choinski and Tuohy 1991).

Before planting each seed was washed briefly in water to remove dung debris and to re‐inspect its testa to confirm infestation state (no significant reclassification occurred). Planting was performed in black, perforated plastic bags (2 L) filled with a mixture of silt and sand (not sterilized) collected in a nearby wadi. Each bag was thereafter soaked in a bucket of water until saturation. The seed was planted in a fingertip‐sized depression and overlaid with either wet soil or minced manure (camel or goat) as given in the randomization table. The amount of manure added was approximately equivalent to 2 goat/1 camel pellet(s).

All bags were watered in the same fashion until saturation and as gently as possible to avoid soil or dung being washed away or seed exposed (if this happened, the seed was carefully reburied). For the first 6 days watering was done twice a day, morning, and afternoon, and thereafter, once a day. After watering each bag was inspected for visible germination, that is, extrusion of the radicle from the soil surface. The last germination was registered on 5. April 2012.

Both the time and date of planting and germination were recorded. To fully utilize the time information, time‐to‐event analyses (aka survival analyses) were performed, using time since planting as the time scale (Collett 2003; Aalen et al. 2008). Seeds that did not germinate within the timeframe of the experiment were treated as censored data and given a censoring time that corresponded to the last day of follow‐up, that is, the last day the seeds were checked for germination. To compare, for instance, the germination rates of seeds with zero, one, or two holes, we used Kaplan–Meier curves combined with the log‐rank test for difference. We used the “1 minus Kaplan–Meier” curves to show the proportion of planted seeds that had germinated on any given day after planting. They are nonparametric in that they assume no particular shape of the germination time distribution, and they correctly handle censoring at end of follow‐up.

Furthermore, to obtain an estimate of the difference in germination, proportional hazards Cox regressions were performed. This provides an estimate of the hazard ratio (HR), that is, the ratio of the intensity of germination in two groups. As factors such as holes and area could not be fully balanced/randomized, the Cox regression was also used for multivariate analyses, adjusting factors for one another in a joint analysis.

As observed for the viability analyses, the germination rate of seeds might depend on what tree they came from; conceivably, seeds from the same tree could share either high or low germination rates. To account for this, we also ran the Cox regressions with a frailty term included. Frailty is a random effect allowing the germination rate to vary among selected trees including their immediate surroundings. We treated *tree ID* as variable for all contexts. Analyses were performed with the survival package in R (Therneau 2014).

## Results

### Seed viability

A total of 16534 seeds were collected (Table 3); of these 19% were intact, 20% had one hole, 19% had two holes or more and the remainder of the seeds were clearly inviable. *Context* of seeds significantly influenced infestation level (*P *=* *2.2e‐16) with seeds collected from dung having much lower odds of being infested (0.33) than seeds from the canopy (11.4) and from the ground (63.3). At the *subcontext* level (*P *<* *2.2e‐16) green pods from either canopy or the ground have significantly lower odds of being infested than seeds from dry pods on the ground (reference), but still have relatively high probability of being infested. Seeds from ovicaprid dung have lower odds of being infested than those from camel dung, and both are significantly less infested than the reference.

**Table 3:** Number of seeds summarized per area, context and subcontext, and infestation status

Area	Code	Context; Subcontext^a^	D/P^b^	S^c^	H0^d^	H1^e^	H2^f^	D^g^
ED	10	G; LS	NA	347	97	125	103	22
RSH	10	G; LS	NA	103	59	19	5	20
WD	10	G; LS	NA	8	5	2	1	0
ED	11	G; LS_DP	NA	371	11	127	175	58
RSH	12	G; GP	7	14	0	0	0	14
WD	12	G; GP	168	523	112	76	89	246
ED	13	G; DP	431	1703	199	449	612	443
RSH	13	G; DP	40	108	9	37	36	26
WD	13	G; DP	502	2059	433	726	383	517
WD	17	G; BlP	773	3101	628	531	350	1592
WD	32	T; GP	575	2372	694	53	38	1587
WD	33	T; DP	1161	4836	176	1061	1249	2350
ED	20	D; C	1227	630	466	59	8	97
RSH	20	D; C	719	58	37	10	2	9
WD	20	D; C	22	1	0	0	0	1
ED	21	D; O	2677	103	93	5	1	4
RSH	21	D; O	5919	155	114	28	8	5
WD	21	D; O	1036	39	24	2	1	12
Sum				16531	3157	3310	3061	7003

a G, Ground; T, tree; D, dung; LS, loose seeds; DP, dry pods; GP, green pods; BlP, Black pods; C, camel; O, Ovicaprid.

b Number of dung pellets (D) or pods (P).

c Number of seeds.

d Number of intact seeds.

e Number of seeds with one entry hole.

f Number of seeds with 2 or more holes.

g Number of clearly inviable seeds or unripe seeds in case of seeds from green pods.

There is a significant regional variation in seed infestation (*P *=* *1.4e‐05). Compared to the RSH, the odds of being infested in the WD are 10 times greater while for the ED they are 2.5 times greater. At the area level, using RSH‐N as reference, seeds from EDN, GK, and UWT have significantly higher odds of being infested. However, these individual areas are not significantly different from RSH‐N when both *area* and *subcontext* are included as variables in the model (*P *<* *0.1, see Table 4). Nevertheless, the area effect as such still has a significant effect in the bivariate model (*P *=* *0.02). The subcontext effect of the univariate model is corroborated in the bivariate model (Table 4).

**Table 4:** Results of logistic regressions with random effects at tree level for subcontext + area effect; OR is the odds ratio of being infested relative to reference category (subcontext = Dry pods on the ground; area = RSH_N). For abbreviations see Table 3. The likelihood ratio test is significant for both the effect of subcontext (*P *<* *2.2e‐16) and area (*P *=* *0.02)

	OR	95% CI	*P*
(Intercept)	14.63	6.24, 34.29	0.000
10	0.62	0.35, 1.09	0.096
11	4.41	1.72, 11.32	0.002
12	0.21	0.11, 0.39	0.000
17	0.80	0.43, 1.51	0.496
20	0.03	0.02, 0.04	0.000
21	0.01	0.01, 0.02	0.000
32	0.09	0.05, 0.16	0.000
33	0.68	0.36, 1.27	0.225
Djara	0.14	0.02, 1.12	0.064
EDC	0.52	0.10, 2.67	0.434
EDN	0.84	0.29, 2.41	0.747
GK	2.51	0.89, 7.09	0.083
RSH_S	5.14	0.19, 142.39	0.334
RSH_W	0.59	0.08, 4.35	0.609
UWT	17.36	0.59, 512.90	0.099

### Germination experiment

**Figure 3:**
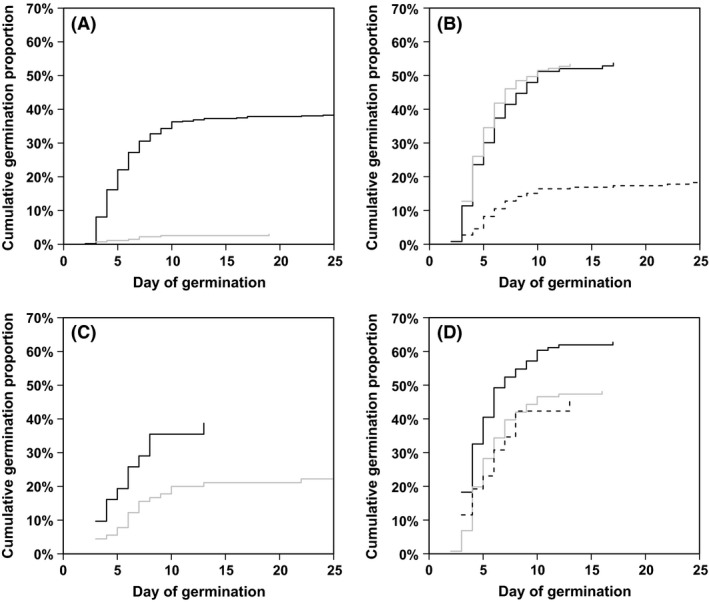
Curves for cumulative germination proportion (1 minus the Kaplan–Meier curve). (A) There is a significant effect on germination success (*P *=* *0) of number of holes (0 = black line; 1 = gray line; see Table 5). (B) There is a significant regional effect on germination success (*P *=* *8.7e‐15; RSH = gray line; ED = black line; and WD = dashed line; see Table 6). (C) Having removed the effect of animal ingestion we still find a strong regional effect on germination success (ED + RSH = black line; WD = gray line; *P *=* *0.07). (D) There is a significant effect (*P *=* *0.008) of ingestion by goat on germination success in RSH + ED (goat = black line; camel = gray line and loose seeds on the ground = dashed line; see Table 7).

Of the 1193 seeds planted 1071 are included in the analyses (122 are excluded because another species than *A. tortilis* germinated). Of these 205 seeds germinated, that is, a 19.1% overall germination. Almost 40% of intact seeds germinated, while less than 2% of infested seeds germinated; that is, of the H1 seeds 3% germinated and of H2 seeds only 0.3% germinated. The number of holes has a significant effect on the germination success (Table 5 and Fig. 3A), and there is no effect of ingestion on germination among infested seeds.

**Table 5:** Germination success expressed as Hazard Ratio (HR) with 95% confidence interval (CI) and *P*‐values, as computed using a Cox regression model with time to germination as time scale. The model compares germination of seeds with one hole (H1) and two holes (H2) to seeds with no holes (reference)

	HR	95% CI	*P*
H1	0.06	0.04, 0.09	1.10E‐14
H2	0.01	0.00, 0.02	7.40E‐07

Due to the very low germination success of infested seeds (*n* = 9) these are excluded from the remainder of the analysis. Germination of intact seeds is significantly lower and slower in the WD than in RSH and ED (Table 6 and Fig. 3B). Less than 20% of the seeds from WD germinated and took up to 10 days to germinate, while the same fraction of seeds only took 4 days in RSH and ED. In RSH and ED, 40% of the intact seeds germinated. Excluding the effect of animal ingestion on germination (testing only intact seeds from the ground) there is still a large but insignificant difference in germination among the regions (Fig. 3C; *P *=* *0.07).

**Table 6:** Germination success expressed as Hazard Ratio (HR) with 95% confidence interval (CI) and *P*‐values, as computed using a Cox regression model with time to germination as time scale. The model compares germination of seeds from regions RSH and WD to seeds from ED (reference)

	HR	95% CI	*P*
RSH	1.03	0.88, 1.21	8.50E‐01
WD	0.27	0.22, 0.33	6.00E‐11

Due to the large difference in germination among regions, the remainder of the analyses treat WD as a separate region from the rest (RSH + ED). There is found no significant difference in germination caused by adding manure from either ovicaprid or camel for either region (WD *P *=* *0.792; ED + RSH *P *=* *0.36). There was also no effect of seed context for either region (WD: ground vs. canopy; *P *=* *0.199; RSH + ED: ground vs. dung; *P *=* *0.090). However, for RSH+ED a significantly higher proportion of seeds from ovicaprid dung (63%) germinated than from either camel dung (48%) or loose seeds (46%) (Fig. 3D; *P *=* *0.008). A Cox regression (Table 7) confirms this result and shows that there is no confounding effect between the variables “manure” and “context”. For the combined region RSH+ED there is found no significant effect of *site* (*P *=* *0.089) or *tribal management* (*P *=* *0.6) on seed germination success.

**Table 7:** Germination success expressed as Hazard Ratio (HR) with 95% confidence intervals (CI) and *P*‐values, as computed from a Cox regression model, using time to germination as time scale. LRT refers to Likelihood Ratio Test. There is no significant effect of manure (reference no manure) on germination when taking into account the context of seed (reference ovicaprid feces). Compared to seeds from ovicaprid feces on any given day after planting, only 64.9% and 61.3% of seeds from camel dung and loose on ground, respectively, germinates. The difference in germination of seeds from ovicaprid and camel feces is significant. LRT shows that the effect of context is significant (*P = *0.025)

	HR	95% CI	*P*	LRT	Loglik
Manure: Camel	0.98	0.80, 1.20	0.920	Manure	−816.18
Manure: Ovicaprid	1.25	1.03, 1.51	0.240		
Context: Camel dung	0.65	0.55, 0.77	0.011	Manure + context	−812.48
Context: Loose on ground	0.61	0.45, 0.84	0.120		

All Cox regressions were run with a frailty term, but this led to only minimal changes in the original parameter estimates. There was no evidence of significant tree‐to‐tree variation in seed germination rate within area/region.

## Discussion

### Seed viability

Some regional differences in the sampled material should be kept in mind when interpreting results. Seedpods from trees' canopies were only sampled from the WD, GK, and UWT. 22% of WD seeds are from green pods and less than 1‰ is from dung. In the RSH we found very few pods on the ground, most probably due to the presence of animals that eat this highly valued fodder source as soon as it is available (Miller 1994a; Rohner and Ward 1999; Andersen et al. 2014).

The overall infestation of seeds sampled in our study area is 81%. Although the variation is significant among trees in focus, we consider this estimate robust and regionally representative because it is based on many samples from a large region and from different contexts. Infestation level is in general significantly influenced by sub‐/context. We find that more older/dry pods are infested than fresher/green ones, as shown by Miller (1996a) and Rohner and Ward (1999). There is a tendency for more dry pods on the ground to be infested than those in the canopy, suggesting that seeds are reinfested on the ground (Miller 1996a; Or and Ward 2003; Ward et al. 2010). Some unripe, but already infested seeds in our material indicate early infestation (Ernst et al. 1989).

The infestation of uningested *A. tortilis* seeds in our region is 84% and comparable to the high levels reported for supsp. *tortilis* and *raddiana* from Arava, Negev, and Sinai (74–99%; Halevy 1974; Rohner and Ward 1999). Other studies including many/repetitive and hence robust measures report infestation values ranging between 5–100% (subsp. *spirocarpa;* Pellew and Southgate 1984), 26–80% (subsp. *raddiana;* Derbel et al. 2007), 19–34%, and 31–68% (subsp. *heteracantha;* Ernst et al. 1989; Miller 1996a).

The overall infestation of ingested seeds (26%) is significantly lower than for uningested seeds, but still about 10 times higher than reported by Coe and Coe (1987) for sheep (2.4%) and Miller (1994b) for wild ungulates (0–3%). We find no difference in proportion of ingested, intact seeds between ovicaprids (78%) and camels (73%). As we did not follow individual seeds' fate we cannot reject the idea that smaller animals chew/destroy a larger proportion of ingested seeds (Miller and Coe 1993; Rohner and Ward 1999). There are only a few studies including ovicaprids and seeds of *A. tortilis* (various subsp.) in digestion experiments, and they show that 9–43% of seeds ingested by goat and 7–10% by sheep remain intact (Ahmed 1986; Miller 1995; Shayo and Uden 1998). No such estimates have been found for dromedary camels.

Geography (*region/area*) has a significant effect on seed viability in our models but is much weaker than that of *context*. At the regional level this is probably related to the skewed sampling of pods from the WD (high infestation) and animal dung from the RSH+ED (low infestation). At the area level, the remaining and significant effect (after removing the effect of subcontext) suggests that there are other geographical factors influencing infestation rate that remain to be identified, although it might be related to temporal and spatial variation in the cycle and distribution of infestation level or at the time of that cycle in which sampling was carried out.

### Germination success

Germination success might conceivably depend on what season the seeds were collected in, and different sites were sampled at different times of the year. However, the experiment was as balanced as possible, meaning that effect estimates of variables such as manure and subcontext should remain valid in spite of this. Furthermore, as site effects were nonsignificant, there is little evidence that sampling time has influenced the germination results.

Overall germination success of infested seeds is 0.3% (H2) and 3% (H1) and significantly lower than for intact seeds (uningested 19%; ingested 60%). Therefore, the hypothesis that recent/partial infestation (1 hole) followed by digestion can have a positive effect on germination rate (Halevy 1974; Coe and Coe 1987; Miller and Coe 1993) is not supported. Ernst et al. (1989) also concluded that cotyledon and radicula damage was too high for this mechanism to be of any advantage. Two factors can have affected our result. Firstly, it is possible that entrance and exit holes have been misidentified as we did not identify bruchid species (size is species specific). However, as there is an effect of 1 versus more than 1 hole this seems to be of minor importance. Secondly, for seeds with only one entry hole we do not know time since infestation and hence the amount of damage to the embryo when it was ingested or germinated.

Of the intact seeds the germination success varied from around 19% (WD, mainly pods) up to ca 60% (ED + RSH, ovicaprid dung). This is the same trend and magnitudes as found in other germination studies of *A. tortilis* (various subsp.) involving ruminants such as gazelles, oryx and goat (e.g., Ahmed 1986; Rohner and Ward 1999), but Reid and Ellis (1995) found only 2–15% germination of seeds in corrals (7 times higher than outside). Shayo and Uden (1998), however, found the opposite tendency (intact, ingested seeds 19‐27% vs. intact seeds 42%).

Our experiment contradicts previous findings that seeds ingested by larger animals have better germination (Miller 1995; Rohner and Ward 1999; Or and Ward 2003; Bodmer and Ward 2002). Seeds from camel dung germinate at the same rate as intact, undigested seeds, and significantly less than seeds from ovicaprid dung. Camels have been proposed as particularly important for conservation of acacias (Rohner and Ward 1999; Bodmer and Ward 2002), but this seems to be a deduction from a body‐mass relationship only and not based on actual germination of ingested seeds. There are also few studies including seeds ingested by ovicaprids. The body mass of dromedary camels (350–620 kg fully grown) and ovicaprids (sheep 55 kg, goat 47 kg; (Lechener‐Doll et al. 1990) are both on the lower end of the range inspected by Miller (1995) where its effect upon seed survival is unclear. A likely reason is that forestomach mean retention time in the dry season (Oct) for small‐sized particles (2/20 mm; seed c. 6 × 4 × 2 mm) is similar for ovicaprids (c. 30/48 h goats; 35/48 h sheep) and camels (c. 30/60 h) (Lechener‐Doll et al. 1990). This suggests that other factors than retention time must explain the positive effect of ovicaprid ingestion.

Although some studies support the hypothesis that dung facilitates seed germination (see references in Miller 1995), we find no positive effect of manure/dung on germination. Coughenour and Detling (1986) concluded that a possible positive effect is not related to nutrients but rather to moisture content of dung as observed in impala middens and in corral soils (Reid and Ellis 1995; Miller 1996b). Others report a negative effect (Miller 1995; Oconnor 1995; Loth et al. 2005), possibly related to dung hardness, fibrousity, and speed of drying (Coe and Coe 1987; Wilson and Witkowski 1998). Camel and ovicaprids have relatively small, dry, and fibrous pellets with expected good moisture holding capacity; however, we observe no effect probably because of regular watering in our experiment. Still, there can be positive secondary dispersal effects as wind might disperse the light droppings of ovicaprids (Ahmed 1986), and during flooding dry dung pellets can float with and redeposit where the water slows down, for example, in small depressions, where infiltration creates optimal moisture conditions for successful recruitment.

Ingestion by herbivores explains the main difference in germination of intact seeds between WD and ED + RSH, but also after excluding this effect twice as many seeds germinate at twice the speed in ED + RSH (*P *=* *0.07). We cannot exclude that some loose seeds sampled from the ground have been spit out during rumination, but we prefer to consider other interpretations as acacia populations in the WD grow in a more extreme environment, are small, isolated and less affected by ruminants. The lower germination in WD indicates a higher proportion of dormant/hard seeds, which might be an adaptation to reduce risk for seedling mortality in a variable/uncertain environment. However, we expect other mechanisms than bet‐hedging (Cohen 1966) to be at play for perennial trees with annual seed output. For other species/genera the proportion of soft and hard seeds may vary at seed maturity (Morrison et al. 1992; Meisert 2002), as also our data might indicate. It has been hypothesized that imbibed, soft seeds are smelling cues for rodents and that plants “benefit from producing dimorphic soft and hard seeds at ratios where the antipredator advantages of hard seeds are balanced by the dispersal benefits gained by producing some soft seeds” (Paulsen et al. 2013, 2014). Although pods of *A. tortilis* have several traits (smell, size, shape, nutrient content) that signify ungulates as main dispersers (Miller and Coe 1993), rodents are important for germination (Miller 1995) and dispersal, both in the presence (15%) and absence (41%) of ungulates (Miller 1994a). Rodents might be more important seed dispersers in the WD than in the ED + RSH because of the historically lower presence of wild and domestic herbivores. Hard seeds might therefore have been oversampled in WD relatively to ED + RSH with subsequently lower germination. Also in a longer term, evolutionary scenario where dispersal increases germination success and hard seeds have higher fitness than soft seeds, it is beneficial to have a lower proportion of soft seeds as humidity decreases (Paulsen et al. 2014). This can be of importance for the variation in soft:hard seed ratios and therefore seed germination we see among regions.

## Conclusions

This study suggests that small stock ingestion has a significantly better effect on germination than does camel ingestion. The consequence for acacia conservation is to acknowledge the positive effect of domestic animals in an ecosystem where wild herbivores are increasingly rare. Having a dispersal agent that removes seeds soon after maturation has been suggested as a Leguminosae trait that may be functional against bruchid destruction (Janzen 1969; Halevy 1974). Traditional herding and tending of trees can act in this way. Shaking, a traditional strategy to feed small stock, removes ripe pods from canopies sooner than otherwise, consequently herded animals will disperse a higher fraction of intact seeds (subcontext effect). Another important factor in traditional herding is the speed and varied pattern of movement which prevents overgrazing and hence is not a threat to postgermination survival (Andersen et al. 2014). Our findings also suggest that soft:hard seed ratios and the effect of rodents on acacia recruitment should be investigated.

## Conflict of Interest

None declared.

## References

[ece31851-bib-0001] Aalen, O. O. , Ø. Borgan , and H. K. Gjessing . 2008 Survival and event history analysis: a process point of view. Springer, New York.

[ece31851-bib-0002] Ahmed, A. E. 1986 Some aspects of dry land afforestation in the sudan with special reference to Acacia‐Tortilis (Forsk) Hayne, *Acacia senegal* Willd and *Prosopis chilensis* (Molina) Stuntz. For. Ecol. Manage. 16:209–221.

[ece31851-bib-0003] Andersen, G. L. , and K. Krzywinski . 2007 Mortality, recruitment and change of desert tree populations in a hyper‐arid environment. PLoS One 2:e208. 17299588 10.1371/journal.pone.0000208 PMC1784067

[ece31851-bib-0004] Andersen, G. L. , K. Krzywinski , M. Talib , A. E. M. Saadallah , J. J. Hobbs , and R. H. Pierce . 2014 Traditional nomadic tending of trees in the Red Sea Hills. J. Arid Environ. 106:36–44.

[ece31851-bib-0005] Ayyad, M. A. , and S. I. Ghabbour . 1985 Hot deserts of Egypt and Sudan Pp. 149–202 *in* Evenari M., Noy‐Meir I., and Goodall D. W., eds. Hot desert and arid shrublands, B. Elsevier, Amsterdam.

[ece31851-bib-0006] Bates, D. , M. Maechler , B. Bolker , and S. Walker . 2014 lme4: Linear mixed‐effects models using Eigen and S4.

[ece31851-bib-0007] Bodmer, R. , and D. Ward . 2002 Frugivory in large mammalian herbivores Pp. 232–260 *in* Danell K., Bergström R., and Duncan P., eds. Large herbivore ecology and ecosystem and conservation. Cambridge Univ. Press, 524 pp.

[ece31851-bib-0008] Boulos, L. 1999 Flora of Egypt. Al Hadara, Cairo.

[ece31851-bib-0009] Bubenzer, O. , A. Bolten , and F. Darius . 2007 Atlas of cultural and environmental change in arid Africa 240 pp Africa praehistorica, Heinrich Barth Institut, Köln.

[ece31851-bib-0010] Choinski, J. S. , and J. M. Tuohy . 1991 Effect of water potential and temperature on the germination of 4 species of African savanna trees. Ann. Bot. 68:227–233.

[ece31851-bib-0011] Coe, M. , and C. Coe . 1987 Large herbivores, acacia trees and bruchid beetles. S. Afr. J. Sci. 83:624–635.

[ece31851-bib-0012] Cohen, D. 1966 Optimizing reproduction in a randomly varying environment. J. Theor. Biol. 12:119–129. 6015423 10.1016/0022-5193(66)90188-3

[ece31851-bib-0013] Collett, D. 2003 Modelling survival data in medical research, 2nd edn Chapman and Hall, Boca Raton, FL, 408 pp.

[ece31851-bib-0014] Coughenour, M. B. , and J. K. Detling . 1986 *Acacia tortilis* seed‐germination responses to water potential and nutrients. Afr. J. Ecol. 24:203–205.

[ece31851-bib-0015] Darius, F. 2013 Wadi Sura in its environmental setting Pp. 70–79 *in* Kuper R., ed. Wadi sura ‐ cave of beasts. Heinrich Barth Institute, Köln.

[ece31851-bib-0016] Derbel, S. , Z. Noumi , K. W. Anton , and M. Chaieb . 2007 Life cycle of the coleopter *Bruchidius raddianae* and the seed predation of the *Acacia tortilis* subsp *raddiana* in Tunisia. C.R. Biol. 330:49–54. 17241947 10.1016/j.crvi.2006.09.003

[ece31851-bib-0017] Ernst, W. H. O. , D. J. Tolsma , and J. E. Decelle . 1989 Predation of seeds of *Acacia tortilis* by insects. Oikos 54:294–300.

[ece31851-bib-0018] Halevy, G. 1974 Effects of gazelles and seed beetles (Bruchidae) on germination and establishment of acacia species. Isr. J. Bot. 23:120–126.

[ece31851-bib-0019] Hobbs, J. J. , K. Krzywinski , G. L. Andersen , M. Talib , R. H. Pierce , and A. E. M. Saadallah . 2014 Acacia trees on the cultural landscapes of the Eastern Sahara. Biodivers. Conserv., 23:2923–2943.

[ece31851-bib-0020] Janzen, D. H. 1969 Seed‐eaters versus seed size number toxicity and dispersal. Evolution 23 1:1–27. 10.1111/j.1558-5646.1969.tb03489.x 28562964

[ece31851-bib-0501] Kyalangalilwa, B. , J. S. Boatwright , B. H. Daru , O. Maurin , M. van der Bank , et al. 2013 Phylogenetic position and revised classification of Acacia s.l. (Fabaceae: Mimosoideae) in Africa, including new combinations in Vachellia and Senegalia. Bot. J. Linn. Soc. 172:500–523.

[ece31851-bib-0021] Lechener‐Doll, M. , T. Rutagwenda , H. J. Schwartz , W. Schultka , and W. V. Engelhardt . 1990 Seasonal changes of ingesta mean retention time and forestomach fluid volume in indigenous camels, cattle, sheep and goats grazing in thornbush savannah pasture in Kenya. J. Agric. Sci. 115:409–420.

[ece31851-bib-0022] Loth, P. E. , W. F. de Boer , I. M. A. Heitkonig , and H. H. T. Prins . 2005 Germination strategy of the East African savanna tree *Acacia tortilis* . J. Trop. Ecol. 21:509–517.

[ece31851-bib-0023] Meisert, A. 2002 Physical dormancy in Geraniaceae seeds. Seed Sci. Res. 12:121–128.

[ece31851-bib-0024] Menne, M. J. , I. Durre , R. S. Vose , B. E. Gleason , and T. G. Houston . 2012 An overview of the global historical climatology network‐daily database. J. Atmos. Ocean Tech. 29:897–910.

[ece31851-bib-0025] Miller, M. F. 1994a The fate of mature African acacia pods and seeds during their passage from the tree to the soil. J. Trop. Ecol. 10:183–196.

[ece31851-bib-0026] Miller, M. F. 1994b Large African herbivores, bruchid beetles and their interactions with acacia seeds. Oecologia 97:265–270. 10.1007/BF00323159 28313938

[ece31851-bib-0027] Miller, M. F. 1995 Acacia seed survival, seed‐germination and seedling growth following pod consumption by large herbivores and seed chewing by rodents. Afr. J. Ecol. 33:194–210.

[ece31851-bib-0028] Miller, M. F. 1996a Acacia seed predation by bruchids in an African savanna ecosystem. J. Appl. Ecol. 33:1137–1144.

[ece31851-bib-0029] Miller, M. F. 1996b Dispersal of Acacia seeds by ungulates and ostriches in an African savanna. J. Trop. Ecol. 12:345–356.

[ece31851-bib-0030] Miller, M. F. , and M. Coe . 1993 Is it advantageous for acacia seeds to be eaten by ungulates. Oikos 66:364–368.

[ece31851-bib-0031] Morrison, D. A. , T. D. Auld , S. Rish , C. Porter , and K. Mcclay . 1992 Patterns of testa‐imposed seed dormancy in Native‐Australian legumes. Ann. Bot. 70:157–163.

[ece31851-bib-0032] Oconnor, T. G. 1995 Acacia Karroo invasion of grassland ‐ environmental and biotic effects influencing seedling emergence and establishment. Oecologia 103:214–223. 10.1007/BF00329083 28306776

[ece31851-bib-0033] Or, K. , and D. Ward . 2003 Three‐way interactions between Acacia, large mammalian herbivores and bruchid beetles ‐ a review. Afr. J. Ecol. 41:257–265.

[ece31851-bib-0034] Paulsen, T. R. , L. Colville , I. Kranner , M. I. Daws , G. Hogstedt , V. Vandvik , et al. 2013 Physical dormancy in seeds: a game of hide and seek? New Phytol. 198:496–503. 23421728 10.1111/nph.12191

[ece31851-bib-0035] Paulsen, R. P. , G. Högstedt , K. Thompson , V. Vandvik , and S. Eliassen . 2014 Conditions favouring hard seededness as a dispersal and predator strategy. J. Ecol. 102:1475–1484. 25558091 10.1111/1365-2745.12323 PMC4277852

[ece31851-bib-0036] Pellew, R. A. , and B. J. Southgate . 1984 The parasitism of *Acacia tortilis* seeds in the serengeti. Afr. J. Ecol. 22:73–75.

[ece31851-bib-0037] R Core Team 2014 R: A language and environment for statistical computing. R Foundation for Statistical Computing, Vienna.

[ece31851-bib-0038] Rabe‐Hesketh, S. , and A. Skrondal . 2012 Multilevel and longitudinal modeling using Stata, 3rd ed. Stata Press, College Station, TX.

[ece31851-bib-0039] Reid, R. S. , and J. E. Ellis . 1995 Impacts of pastoralists on woodlands in South Turkana, Kenya ‐ livestock‐mediated tree recruitment. Ecol. Appl. 5:978–992.

[ece31851-bib-0040] Rohner, C. , and D. Ward . 1999 Large mammalian herbivores and the conservation of arid Acacia stands in the Middle East. Conserv. Biol. 13:1162–1171.

[ece31851-bib-0041] Shayo, C. M. , and P. Uden . 1998 Recovery of seed of four African browse shrubs ingested by cattle, sheep and goats and the effect of ingestion, hot water and acid treatment on the viability of the seeds. Trop. Grasslands 32:195–200.

[ece31851-bib-0042] Therneau, T. 2014 A Package for Survival Analysis in S.

[ece31851-bib-0043] Ward, D. , and C. Rohner . 1997 Anthropogenic causes of high mortality and low recruitment in three *Acacia* tree taxa in the Negev desert, Israel. Biodivers. Conserv. 6:877–893.

[ece31851-bib-0044] Ward, D. , I. Musli , K. Or , T. Gbenro , and O. Skutelsky . 2010 Bruchid seed infestation and development time in three host species of Acacia (Coleoptera, Bruchidae). Zool. Middle East 51:95–103.

[ece31851-bib-0045] Wilson, T. B. , and E. T. F. Witkowski . 1998 Water requirements for germination and early seedling establishment in four African savanna woody plant species. J. Arid Environ. 38:541–550.

